# Coupling analysis of population aging and economic growth with spatial-temporal variation: a case study in China

**DOI:** 10.1186/s12939-022-01711-7

**Published:** 2022-08-13

**Authors:** Shaobin Wang, Zhoupeng Ren, Zhuoyao Xiao, Na Wang, Hao Yang, Haixia Pu

**Affiliations:** 1 Institute of Geographic Sciences and Natural Resources Research, Chinese Academy of Sciences, Beijing, China; 2 State Key Laboratory of Resources and Environmental Information System, Institute of Geographic Sciences and Natural Resources Research, Chinese Academy of Sciences, Beijing, China; 3 School of Geography, University College Dublin, Dublin, Ireland; 4 School of Applied Economics, Renmin University of China, Beijing, China; 5 Beijing Academy of Social Sciences, Beijing, China; 6 Population Development and Policy Research Center, Chongqing Technology and Business University, Chongqing, China

**Keywords:** Population aging, Economic development, Coupling and coordination model, Standard deviational ellipse, Sustainable development

## Abstract

**Background:**

China now faces an increasingly aging society which may exert economic pressure in the long run. This study illustrates the spatial pattern and evolution of population aging and economic development in China. The coupling coordination degree of population aging and economic development at the national and provincial levels are calculated and demonstrated, and the spatial patterns and characteristics are investigated.

**Methods:**

This paper presents a coupling analysis of the elderly population rate (EPR) and per capita Gross Regional Product (GRP_pc_) in China by using the coupling and coordination model. Further, the spatial pattern and evolution of population aging and economic development are investigated based on the standard deviational ellipse. The collected data is at the level of provincial administrative units in mainland China covering the period 2002 to 2020.

**Results:**

The results reveal the spatial difference in the coupling and coordination degree between EPR and GRP_pc_ across provinces. The eastern coastal areas are higher than the central and western regions of China. The orientation and directions of EPR are more than GRP_pc_, indicating that the polarization in population aging is more severe than economic development. Significant positive correlations between coupling coordination degree and sustainable competitiveness are detected.

**Conclusions:**

Policymakers should fully consider regional differences and sustainable development in policy formulation of China. The western and northeastern provinces should be given priority in the regional sustainable development plan. At the same time, the coordination between population aging and economic development also requires to be examined especially.

## Introduction

China is not only the country with the most population globally, but it is also one of the countries with the fastest aged population, which presents severe challenges from a rapidly aging society [1]. The fifth national census in 2000 showed that the number of people aged 60 and older had already reached 126 million and constituted 10% of the total population in China, indicating the coming of an aging society [2]. According to the seventh national census of China in 2020, 13.5% were over 65 years old. Nevertheless, this rate was only 8.87% based on the sixth national census in 2010. Thus, it can be seen that the population aging in China has accelerated growth since the beginning of the twenty-first century [3].

China has achieved substantial economic development success since the reform and opening policy in 1978, which has become an upper-middle-income country since 2020, as defined by the World Bank. For one thing, rapid economic development plays a crucial role in raising life expectancy, especially in developing countries [4–6]. For another, many studies have expressed concern and even alarm about population aging impacts on economic development in China [7–9]. Thus, the relationship between population aging and economic development has gradually become the focus of the academic community. For instance, the negative impact of population aging on economic development has been widely discussed and acknowledged [10–16]. Notably, in the long run, population aging may exert economic pressure on providing services for the growing number of the elderly [17, 18]. Several studies revealed the impacts of population aging on the sustainability of social security systems and national economic growth, highlighting the demand to link population aging with city competitiveness [19] and sustainable development [20–22].

Furthermore, China has a vast territory with obvious differences in the aged population rate [23–28] and economic development in space [29–31]. Prior studies showed that the evolution trend of the overall pattern of economic development and population in China had been probed, indicating a similar movement trend of the economy and the total population with a southwest forward movement trend [32, 33]. The variation trend demonstrates that China’s population and economic development gaps are increasingly salient in the south and north [34]. Another study investigated the variation in spatial-temporal distribution between population aging and economic development, indicating an increase in the economic burden of elderly care in northern China [35]. Therefore, it is imperative to understand the spatial-temporal relations and variation of population aging and economic development.

However, there are still two gaps in the current research. First, most previous studies have tended to the spatial distribution of population aging indicators or economic development separately. Consequently, few studies considered the coupling relationship between population aging and economic growth in China based on a spatial analysis framework. Meanwhile, the spatial visualization of the relationship has also been unexplored. Second, the spatial-temporal variation and difference in the coupling relationship between these two systems and their implications for sustainable development have been unclear in China. Hence, it is of great significance to dynamically display the spatiotemporal characteristics of the coupling and coordination degree between population aging and economic development in China. Further, given the scientific and policy implications, the in-depth investigation of their relations to sustainable development is also crucial. Nevertheless, a quantitative assessment of the relations between these two systems is not a simple assignment because of the links’ spatial differences. Fortunately, the coupling and coordination model (CCDM) method allows us to investigate the degree of coupling — the interaction among two or more systems. Since achieving coordinated development is essential for sustainability, concentrating on this topic is becoming increasingly meaningful [36]. Furthermore, standard deviational ellipse (SDE) modeling provides a summary descriptor of the spatial distribution of points, which is superior for spatial data investigation [37].

Accordingly, to fill the gaps in the previous research, this study mainly investigates the coupling and coordination degree between population aging and economic development in China. First, SDE is performed to illustrate these two systems’ spatial distribution and evolution patterns in China. Then, the dynamic evolution is evaluated by using CCDM, and the spatial-temporal variation is probed. The relations between the coupling coordination degree and sustainable competitiveness are explored as well. We collected panel data on population aging and economic indicators in China at the provincial level from 2002 to 2020 to achieve these aims. This study can contribute to the theoretical and existing literature on population aging and economic growth. This paper could also offer implications for achieving regional sustainable development in a coming aging society in China.

The rest of the study is organized as follows. Section 2 describes the data and methods used to assess the coupling analysis of population aging and economic growth based on CCDM and SDE modeling. In the following sections 3 and 4, we present and discuss our main findings while highlighting the limitations and directions for further study. Finally, the conclusions of the current study and some policy suggestions are provided in section 5.

## Data and methods

### Data sources

The key demographic data for 2020 have been released by the National Bureau of Statistics of China based on the seventh national census. Hence, in this paper, the data on the provincial population with the age composition in 2020 can be obtained (http://www.stats.gov.cn/english/PressRelease/202105/t20210510_1817190.html). Further, demographic data in 2010 are from the sixth national census of China [38]. Demographic data in 2005 and 2015 are from the 1% National Population Sample Survey of China, and data in other years are from the 1‰ National Population Sample Survey of China. The economic indicators are from the *China Statistical Yearbooks*. Data on sustainable competitiveness at the provincial level in 2020 are from the *Annual Report on China’s Urban Competitiveness (No. 18)* released by the National Academy of Economic Strategy, Chinese Academy of Social Sciences (http://gucp.cssn.cn/yjcg/zcjy/202010/t20201027_5202025.shtml). Because the complete and continuous data set can be obtained since 2002, the collected data cover 31 provincial administrative units (including province-level autonomous regions and municipalities) in mainland China with the period 2002 to 2020. Data from Hong Kong, Macau, and Taiwan are not included in this study due to their non-availability.

### Indicators

#### Elderly population rate

The elderly population is commonly defined as 65 years old and over [39]. The elderly population rate (EPR) has been widely applied to reflect the population aging level [35, 40–42]. In this paper, the population aged 65 and above (% of the total population) is calculated as the primary indicator to measure population aging on the provincial scale in China.

#### Gross regional product

Gross regional product (GRP) mainly measures created value through regional production units in the regional economy (e.g., a state, province, or district) [43]. Per capita Gross Regional Product (GRP_pc_) is widely used for the welfare of households approximately within a region. Further, the association between population aging and economic development has been extensively conducted at the country and global levels [27, 44, 45]. Thus, in this study, GRP_pc_ is collected at the provincial level in China to reflect the regional economic development level.

#### Sustainable competitiveness

Compared to economic competitiveness in the short run, sustainable competitiveness is a composite indicator to evaluate the competitiveness level in the long run, which includes six dimensions: knowledge innovation, harmonious society, ecological-environment, culture, whole domain, openness, and further based on twenty-four secondary indicators in specific [46]. Therefore, the sustainable competitiveness of each province in China can be compared with the coupling coordination degree of population aging and economic growth to demonstrate the further implication of the sustainable development potential in this study.

### Methodology

#### Standard deviational ellipse model

**Fig. 1:**
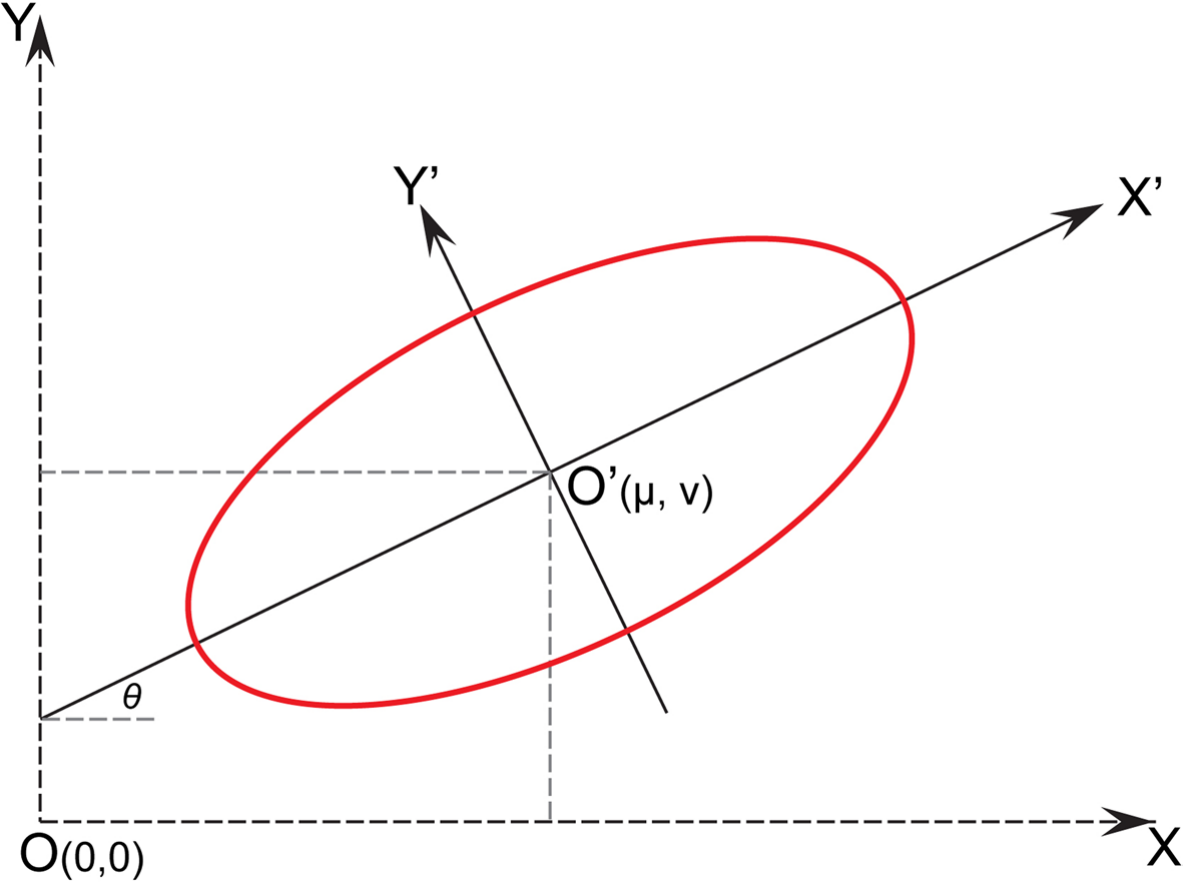
Schematic diagram of the standard deviational ellipse

The standard deviational ellipse (SDE) model has been widely used in spatial assessment, such as air pollution [47], avian influenza [48], waste management [49], industry development [50], etc. This technique computes the standard distances in two directions. It displays the calculation results in an ellipse, intuitively describing the dynamic trends of research elements in space as indicated by a standard planar coordinate system (*X, Y*). The new coordinate system (*X’, Y′*) is formed by a rotation axis according to tan*θ* (Fig. 1), which sets the average midpoint of all points (*μ, ν*) as an origin point. *μ* and *ν* are the average *x* and *y* coordinate values of all points, which can be calculated based on the Eqs. (1–2), respectively.

1 $$\mu =\frac{\sum_{i=1}^n{x}_i}{\mathrm{n}}$$

2 $$\nu =\frac{\sum_{i=1}^n{y}_i}{\mathrm{n}}$$

The long axis (SDE_x_) and minor axis length (SDE_y_) are calculated by Eqs. (3–4).

3 $${SDE}_x=\sqrt{\frac{\sum_{i=1}^n{\left({x}_i-\mu \right)}^2}{n}}$$

4 $${SDE}_y=\sqrt{\frac{\sum_{i=1}^n{\left({x}_i-\upsilon \right)}^2}{n}}$$

In sum, the SDE modeling can describe the study indicators’ centrality, distribution, and orientation. Specifically, the basic parameters of SDE modeling include the mean center, long axes, minor axes, and azimuth. The long and minor axes of the ellipse denote the directions and scope of the data distribution and the dispersion degree (Fig. 1). Hence, this method is selected to investigate the spatial evolution of population aging and economic development. The modeling was performed by using ArcGIS (version 10.2).

#### Coupling and coordination degree model

The concept of coupling, which was originally identified in physics, is defined as a phenomenon in which two or more systems affect each other [51]. The coupling and coordination degree model (CCDM) is a method to explore the coupling coordination degree among systems, which has been widely applied in the interrelationship assessment such as urbanization and atmospheric environment [52], urbanization and energy-environment efficiency [53], city-industry integration development [54], urbanization and eco-environment [55], international trade and carbon emission [56], urbanization and green development [57]. This paper introduces the CCDM to explore the interrelationship between population aging and economic development. Firstly, the coupling degree (*C*) is calculated as follows [52]:

5 $$C=\sqrt{\frac{EPR\times {GRP}_{pc}}{{\left(\frac{EPR+{GRP}_{pc}}{2}\right)}^2}}$$

In Eq. (5), *C* represents the coupling degree between population aging and economic development. Then, the CCDM is performed to calculate the coordination relationship as follows:

6 $$D=\sqrt{C\times T}$$

7 $$T=\alpha EPR+\beta {GRP}_{pc}$$

In Eqs. (6–7), *D* indicates the degree of coupling coordination between population aging and economic development. The range of *D*-value is [0, 1]. The higher *D*-value represents, the higher the coupling degree. *T* is the comprehensive coordinating index to reflect the contribution of the integrated synergy of population aging and economic development. Both *α* and *β* are weights to be determined. In this study, these two indicators are of the same importance, so the values for *α* and *β* are equivalent, i.e., *α* = *β* = 0.5. The coupling coordination degree is divided into five levels (Table 1) [53]. A high coupling state means the economic development and population aging is in a good coordination state, while a low uncoupling state indicates poor levels of coordination.

**Table 1:** Classification of the coupling coordination degree (*D*-value) between population aging and economic growth

*D*-value	Coupling Level	Coordination Status
[0.8 ~ 1.0]	High coupling	Good coordination
[0.6 ~ 0.8)	Moderate coupling	Coordination
[0.4 ~ 0.6)	Low coupling	Basic coordination
[0.2 ~ 0.4)	Moderate uncoupling	Imbalance
[0 ~ 0.2)	Severe uncoupling	Serious imbalance

#### Spatial characteristic analysis

Moran’s *I* is widely used to measure the general spatial autocorrelation degree of attribute variables [58], which can be calculated as follows [59]:

8 $$I=\frac{n\times \sum_i^n\sum_j^n{W}_{ij}\left({x}_i-\overline{x}\right)\left({x}_j-\overline{x}\right)}{\sum_i^n\sum_j^n{W}_{ij}\times \sum_i^n{\left({x}_i-\overline{x}\right)}^2}$$

In Eq. (1), *x* represents the selected indicators (*D*-value, i.e., coupling coordination degrees of population aging and economic development) in this paper; $\overline{x}$ is the mean of *x*; *n* refers to the number of spatial units indexed by *i* and *j*; *W*_*ij*_ refers to an (*n* × *n*) weight matrix depicting the spatial relationship between the selected indicator and its surrounding values based on the queen criterion. Then, *Z*-scores are used to test Moran’s *I* values. The global spatial autocorrelation is detected based on Geoda (version 1.14.0).

#### Coefficient of variation

The coefficient of variation (CV) is commonly used to measure whether the dispersion degree of the study indicators decreases or not during the study period. CV is calculated as follows:

9 $${CV}_{i,t}=\frac{\sqrt{\frac{1}{n}{\sum}_{i=1}^n{\left({x}_{i,t}-{\overline{x}}_{i,t}\right)}^2}}{{\overline{x}}_{i,t}}$$

In Eq. (9), *i* and *t* refer to the province and year, respectively. *n* denotes the total number of provinces. *x* represents the indicator of EPR and GRP_pc_. $\overline{x}$ is the mean value of the indicators *i* in year *t*. The higher the level of CV is, the higher the measured value of dispersion degree is, and vice versa.

## Results

### Spatial patterns and evolution of population aging and economic development

The population aging, economic development, and sustainable competitiveness of each province in China in 2020 show an overall picture (Table 2). The EPR in each province exceeds 7%; the definition of a country or region can be considered an aging society by the United Nations, except Tibet. Meanwhile, huge differences across provinces can also be seen. The eastern provinces, such as Liaoning, Jiangsu, Shanghai, and several southwestern provinces, such as Sichuan and Chongqing, have EPR higher than 15%, indicating a higher aging level. Similarly, the GRP_pc_ also presents great provincial differences in that eastern provinces show higher economic levels than western provinces (Table 2).

**Table 2:** Basic description of population aging, economic development, and sustainable competitiveness of each province in China, 2020

Province	EPR (%)	GRP_pc_ (RMB yuan)	*D*-value	Sustainable competitiveness
Beijing	13.30	164,904.00	0.89	0.73
Tianjin	14.75	101,570.20	0.79	0.58
Hebei	13.92	48,528.06	0.52	0.26
Shanxi	12.90	50,555.97	0.52	0.20
InnerMongolia	13.05	72,185.49	0.65	0.15
Liaoning	17.42	58,967.29	0.65	0.22
Jilin	15.61	51,140.65	0.57	0.20
Heilongjiang	15.61	43,009.30	0.48	0.13
Shanghai	16.28	155,605.90	0.95	0.77
Jiangsu	16.20	121,205.20	0.87	0.44
Zhejiang	13.27	100,070.30	0.75	0.38
Anhui	15.01	63,382.59	0.64	0.22
Fujian	11.10	105,690.40	0.71	0.32
Jiangxi	11.89	56,853.90	0.55	0.23
Shandong	15.13	72,028.79	0.69	0.35
Henan	13.49	55,348.24	0.57	0.27
Hubei	14.59	75,223.44	0.69	0.26
Hunan	14.81	62,881.44	0.64	0.24
Guangdong	8.58	87,896.78	0.57	0.35
Guangxi	12.20	44,201.28	0.45	0.17
Hainan	10.43	54,878.11	0.50	0.29
Chongqing	17.08	78,001.70	0.75	0.44
Sichuan	16.93	58,080.52	0.64	0.21
Guizhou	11.56	46,228.13	0.46	0.24
Yunnan	10.75	51,942.97	0.49	0.14
Tibet	5.67	52,157.01	0.19	–
Shaanxi	13.32	66,234.56	0.63	0.22
Gansu	12.58	36,038.21	0.28	0.08
Qinghai	8.68	50,741.76	0.42	0.24
Ningxia	9.62	54,432.02	0.48	0.13
Xinjiang	7.76	53,370.71	0.40	0.28

**Fig. 2:**
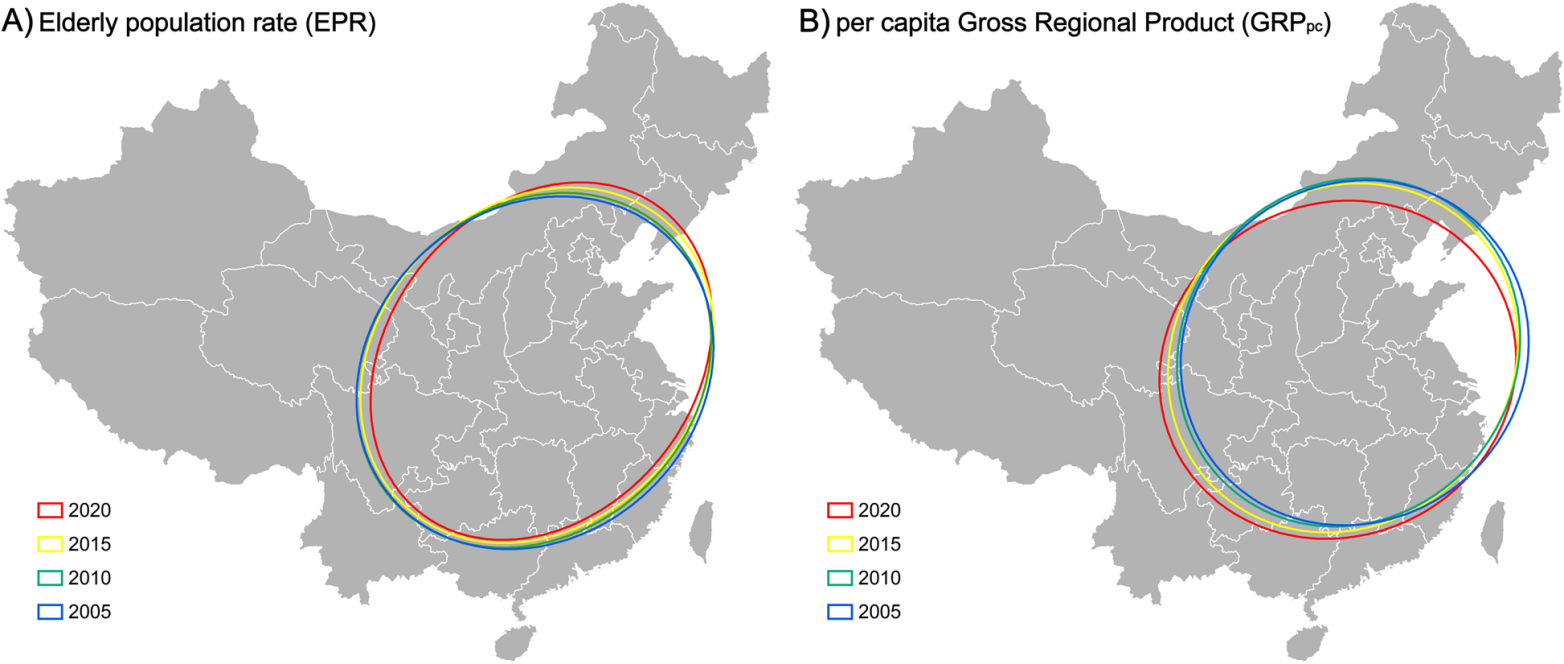
Spatial distribution in the standard deviation ellipse of population aging and economic growth in China in selected years with five-year intervals

Further, the spatial evolution trend of EPR and GRP_pc_ in mainland China can be illustrated from 2002 to 2020 at the provincial level according to the visualization results of the standard deviation ellipse analysis (Fig. 2) and the parameter table of the standard deviation ellipse (Table 3). Several spatial features can be obtained based on SDE modeling.

**Table 3:** SDE parameters of population aging and economic growth from 2002 to 2020

Year	EPR					GRP_pc_				
	Shape Area (10^4^km^2^)	XStdDist (km)	YStdDist (km)	Rotation (^o^)	Y-axis/X- axis	Shape Area (10^4^km^2^)	XStdDist (km)	YStdDist (km)	Rotation (^o^)	Y-axis/X-axis
2020	344.98	931.32	1179.16	39.35	1.27	349.09	1010.98	1099.18	64.80	1.09
2019	352.15	955.87	1172.74	44.45	1.23	345.84	1013.01	1086.78	72.91	1.07
2018	346.57	946.59	1165.49	44.07	1.23	347.06	1015.00	1088.47	74.31	1.07
2017	351.50	958.63	1167.22	44.03	1.22	347.39	1009.01	1095.96	51.74	1.09
2016	353.01	961.97	1168.15	42.28	1.21	348.25	1008.45	1099.28	46.69	1.09
2015	357.91	970.31	1174.18	44.18	1.21	352.60	1010.88	1110.33	45.89	1.10
2014	356.10	966.21	1173.22	44.57	1.21	355.47	1016.40	1113.30	46.11	1.10
2013	353.16	967.56	1161.91	44.34	1.20	353.93	1012.73	1112.49	44.46	1.10
2012	355.63	981.41	1153.50	47.27	1.18	351.56	1008.79	1109.34	41.83	1.10
2011	353.86	969.24	1162.18	45.64	1.20	348.50	1006.27	1102.45	39.97	1.10
2010	359.59	980.93	1166.92	45.01	1.19	345.30	1005.14	1093.56	39.99	1.09
2009	363.85	992.06	1167.50	49.69	1.18	341.59	997.98	1089.59	39.37	1.09
2008	368.58	1000.80	1172.34	48.40	1.17	344.38	1007.19	1088.42	41.59	1.08
2007	367.16	1000.99	1167.63	48.22	1.17	344.85	1011.20	1085.58	43.25	1.07
2006	363.95	996.95	1162.11	49.71	1.17	348.11	1019.49	1086.95	48.03	1.07
2005	361.23	995.36	1155.26	47.34	1.16	347.60	1019.12	1085.74	48.93	1.07
2004	362.27	998.37	1155.07	51.69	1.16	349.16	1021.29	1088.29	51.28	1.07
2003	356.65	989.64	1147.20	48.58	1.16	353.39	1026.99	1095.36	51.55	1.07
2002	364.34	1007.68	1150.95	52.55	1.14	355.37	1025.89	1102.67	50.06	1.07

First, SDEs of EPR in China over the study period show obvious orientation and directions according to the ratio of the Y-axis and X-axis (Table 3). While the ratios of GRP_pc_ are lower than those of EPR. The ratio is close to 1 of GRP_pc_ during the period, indicating that the long axis is nearly equal to the short axis. SDEs demonstrate that the orientations and directions of EPR are more evident than GRP_pc_. The EPR shows a direction from southwest to northeast with an anticlockwise rotation. In comparison, the SDE of GRP_pc_ is nearly a circle with lower ratios of Y-axis/X-axis with a clockwise rotation and weaker directions than EPR.

Second, the Y-axis and X-axis depict these two indicators’ degrees of clustering or dispersion. The major standard ellipse axis can reflect the element ranges of spatial distribution. For EPR, the Y-axis length increases over time. The opposite result is observed in the X-axis, indicating the strengthening of the directional trend and decrease of the dispersion degree. For GRP_pc_, the shortening of the two axes of the ellipse illustrates a spatial aggregation tendency and even spatial changes over time.

Third, a noticeable difference in rotation values variation can be seen between EPR and GRP_pc_. The EPR shows a downward trend from 52.55^°^ in 2002 to 39.35^°^ in 2020, while the GRP_pc_ shows an upward trend from 50.06^°^ in 2002 to 64.80^°^ in 2020. Further, the rotation values of EPR are higher than those of GRP_pc_ from 2002 to 2012, and after 2012, the rotation values of EPR become lower than those of GRP_pc_. Thus, the rotation of these two indicators’ distribution orientation and directions show an opposite trend during the study period.

Last, the ellipse’s size reflects the spatial concentration of population aging and economic growth indicators. Both EPR and GRP_pc_ show a declining trend in the shape area of the standard deviation ellipse. It further indicates the increasing trend of the spatial concentration of these two indicators during the study period in China.

### Overall coupling coordination degrees of population aging and economic development

**Fig. 3:**
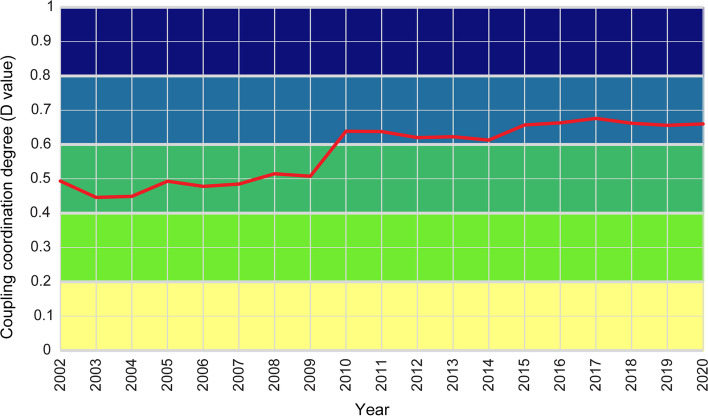
Temporal variation in coupling coordination degree between population aging and economic growth in China, 2002–2020

In this section, the coupling and coordination degrees of EPR and GRP_pc_ were calculated at the national level in China from 2002 to 2020 by using CCDM. The overall variation of coupling coordination degree between population aging and economic growth indexed by *D*-values at the national level was visualized during the study period (Fig. 3). Several key points can be obtained based on the overall coupling coordination analysis. The coupling coordination degree at the national level indicates an overall increasing trend during the study period, rising from 0.58 in 2002 to 0.68 in 2020. It demonstrates that the coupling level between population aging and economic growth changed from low to moderate coupling from 2002 to 2020. Furthermore, this trend can be divided into two stages. From 2002 to 2009, the coupling coordination degree was relatively low, and the two systems (i.e., population aging and economic growth) displayed only a low level of coupling coordination. From 2009 to 2020, EPR and GRP_pc_ were in a moderate coupling stage.

### Spatial characteristics of coupling coordination degree

**Fig. 4:**
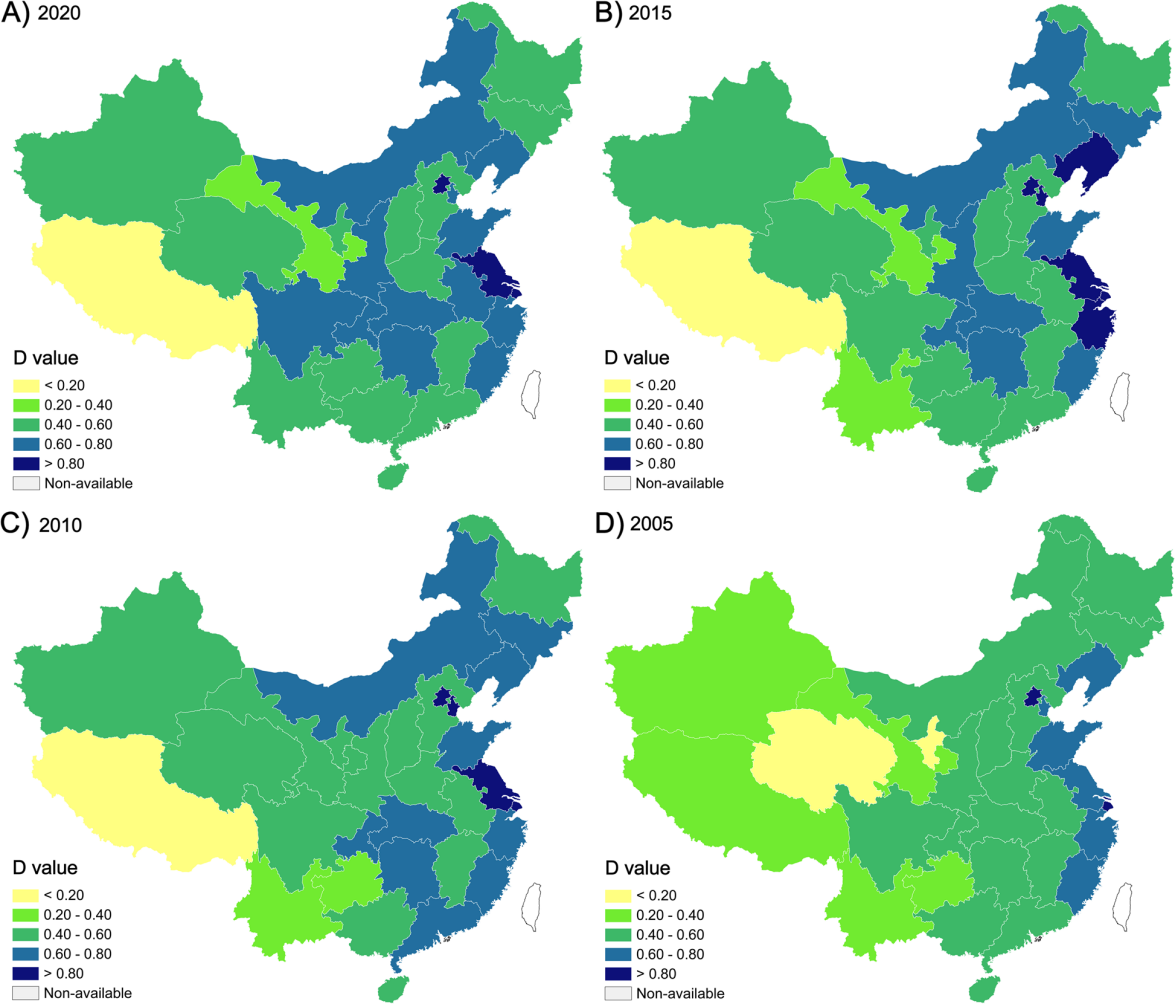
The spatial distribution of the coupling coordination degree (*D*-value) between population aging and economic growth in China in selected years with a five-year interval

To analyze the evolution of the coupling coordination level between population aging and economic growth at the provincial level in China, 2005, 2010, 2015, and 2020 are selected as cross-sectional years during the research period. Coupling coordination degrees at the provincial level in China are then classified and visualized (Fig. 4). Several points can be obtained. First, the apparent difference in *D*-values between the eastern and western regions in China can be seen. The eastern provinces such as Jiangsu, Shanghai, Zhejiang, Shandong, Fujian, and Beijing always exhibit higher *D*-values (> 0.6), indicating moderate coupling or high coupling. While western provinces, such as Tibet, Qinghai, Yunnan, and Gansu, show the lowest *D*-values in China, indicating low coupling or moderate uncoupling. Second, most provinces, such as Ningxia and Guizhou, present an increasing trend of *D*-values, with average annual growth rates of 5.41and 4.08%, respectively, the top two provinces in China. In contrast, Shanghai, Guangdong, Xinjiang, Gansu, and Tibet show a decreasing trend, and Tibet shows the lowest negative growth rate of − 3.62%. Other provinces show slight decreasing rates during the study period. In the process of population aging and economic growth, the degree of coupling and coordination between these two systems has shown apparent regional differentiation.

In addition, spatial autocorrelation indexed by Moran’s *I* was employed to investigate the spatial patterns of *D*-values and revealed spatial dependency across provinces in China from 2002 to 2020 (Table 4). Two main points of spatial characteristics can be demonstrated. First, significant positive autocorrelations can be detected in *D*-values, indicating clustered spatial patterns rather than dispersed or random distribution. Second, the temporal variation of Moran’s *I* statistics shows a trend that firstly decreased and then increased. The lowest value of Moran’s *I* statistics was in 2014, and it showed an inclining trend then. Moreover, CV values of the coupling coordination degree were calculated (Table 4). A stable declining trend of the CV values can be seen during the study period from 0.35 in 2002 to 0.28 in 2020. It indicates a decreasing trend in the dispersion levels of coupling coordination degree across provinces.

**Table 4:** Moran’s *I* with *p-*values and CV of *D*-values in China from 2002 to 2020

Year	Global spatial autocorrelations			Coefficient of variation
	Moran’ s *I*	*Z-score*	*p-*value	
2020	0.317	3.240	< 0.001	0.28
2019	0.336	3.417	< 0.001	0.29
2018	0.342	3.467	< 0.001	0.29
2017	0.354	3.565	< 0.001	0.30
2016	0.327	3.321	< 0.001	0.30
2015	0.329	3.318	< 0.001	0.31
2014	0.255	2.645	0.008	0.33
2013	0.300	3.078	0.002	0.30
2012	0.285	2.940	0.003	0.29
2011	0.290	2.984	0.003	0.28
2010	0.345	3.497	< 0.001	0.29
2009	0.328	3.318	< 0.001	0.36
2008	0.326	3.307	< 0.001	0.36
2007	0.347	3.520	< 0.001	0.36
2006	0.327	3.374	< 0.001	0.34
2005	0.358	3.627	< 0.001	0.36
2004	0.347	3.589	< 0.001	0.37
2003	0.356	3.670	< 0.001	0.39
2002	0.335	3.473	< 0.001	0.35

**Fig. 5:**
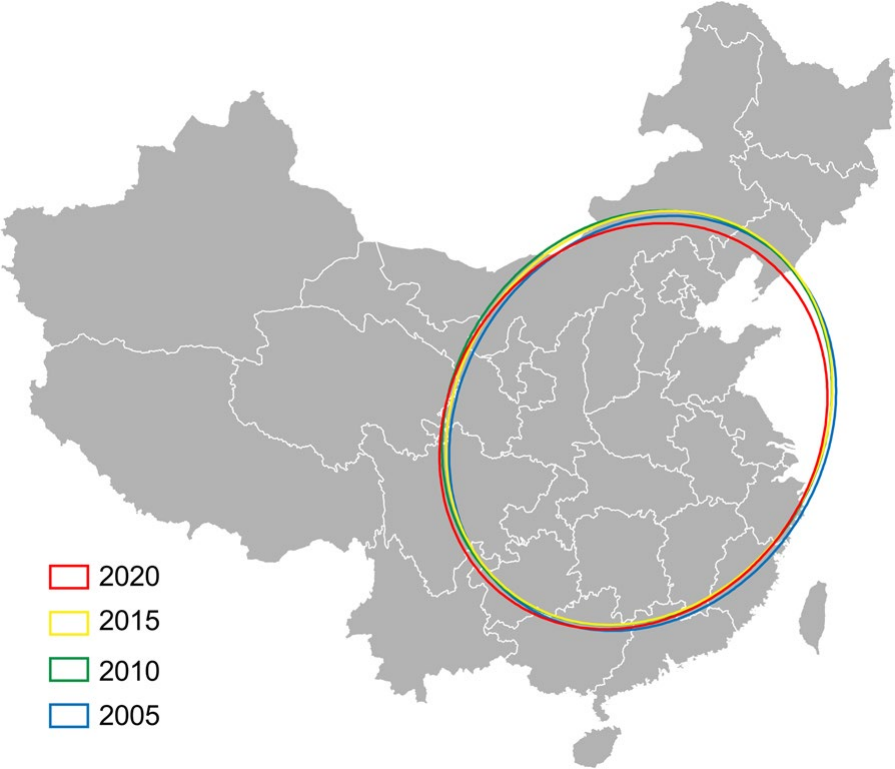
Spatial distribution in the standard deviation ellipse of coupling coordination degree in China in selected years with a five-year interval

Additionally, the SDE model was applied to visually express spatial distribution and dynamic evolutionary processes of the coupling coordination degree indexed by *D*-value (Fig. 5). First, the shape area of the standard deviation ellipse shows a declining trend, which indicates the increasing trend of the spatial concentration of the coupling coordination degree of population aging and economic growth (Table 5). Second, the *Y*-axis length exhibits a decreasing trend over time. A similar result can be observed in the *X*-axis, indicating a spatial aggregation tendency and spatial changes over time. Third, the ratio of the *Y*-axis and *X*-axis shows relative stability during the study period, indicating the orientation and directions did not change obviously. It is consistent with the change of rotation values, which shows a stable trend with fluctuations around the annual average value of 36.83^°^. It demonstrates a stable southwest-northeast distribution pattern based on SDE modeling.

**Table 5:** SDE parameters of coupling coordination degree (*D*-values) from 2002 to 2020

Year	Shape Area (10^4^km^2^)	XStdDist (km)	YStdDist (km)	Rotation (^o^)	Y-axis/X- axis
2020	333.10	953.26	1112.34	36.67	1.17
2019	332.84	969.36	1093.01	41.18	1.13
2018	329.31	960.78	1091.08	39.87	1.14
2017	333.92	950.22	1118.65	35.26	1.18
2016	339.22	960.82	1123.87	33.43	1.17
2015	337.88	951.93	1129.89	33.24	1.19
2014	339.26	958.98	1126.14	32.99	1.17
2013	339.60	960.79	1125.14	33.46	1.17
2012	340.85	969.39	1119.26	34.94	1.15
2011	344.18	970.14	1129.33	35.25	1.16
2010	342.34	965.73	1128.43	33.60	1.17
2009	323.04	909.37	1130.81	33.90	1.24
2008	346.16	968.67	1137.55	35.77	1.17
2007	348.78	973.77	1140.16	36.93	1.17
2006	355.02	989.25	1142.40	41.86	1.15
2005	339.27	954.25	1131.77	33.12	1.19
2004	352.93	989.63	1135.25	42.81	1.15
2003	337.53	948.34	1132.98	36.71	1.19
2002	369.46	1021.66	1151.16	48.79	1.13

### Relations between coupling coordination degree and sustainable competitiveness

**Fig. 6:**
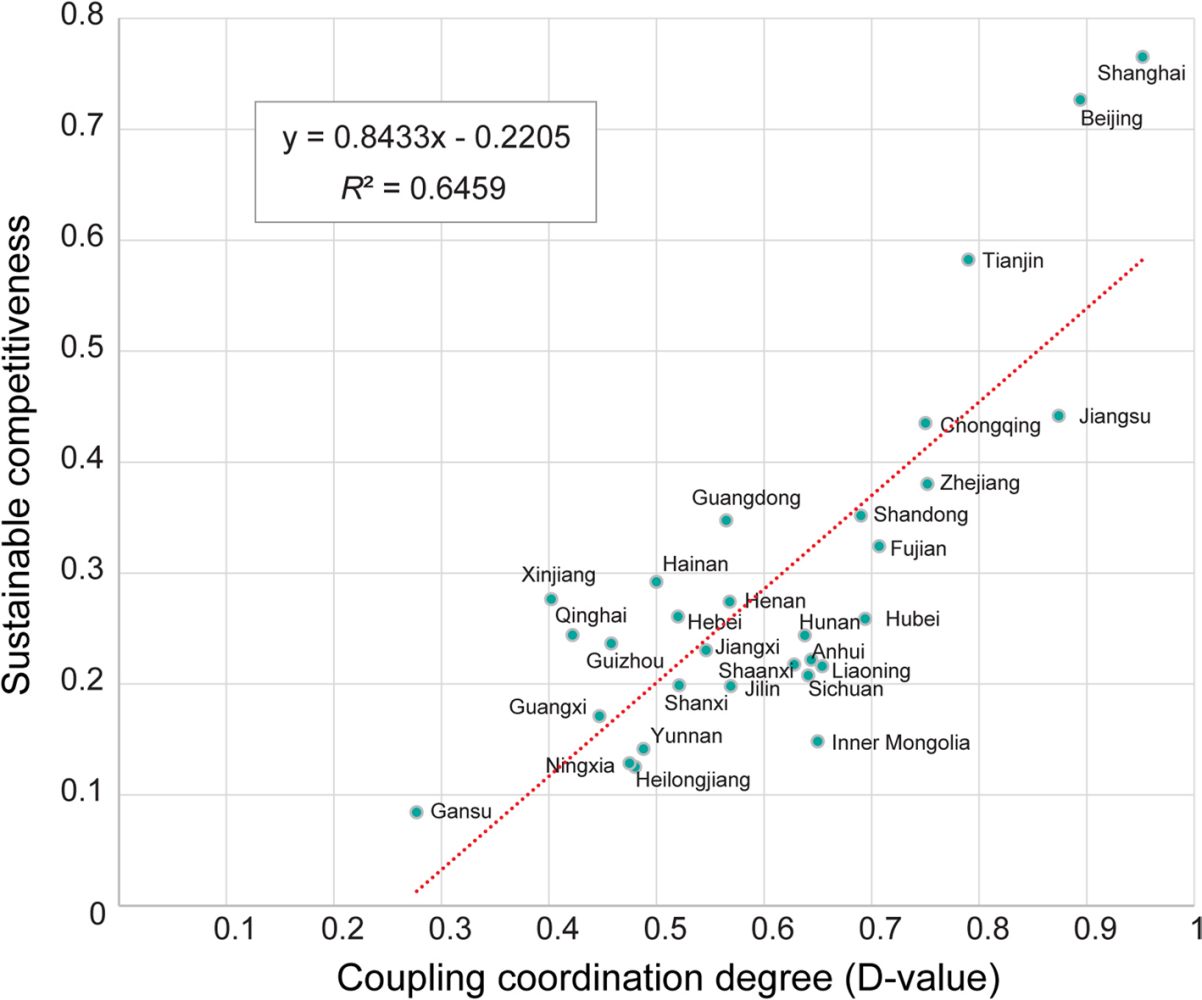
Scatter diagram and fitting curve of coupling coordination degree (indexed by *D*-value) and sustainable competitiveness at the provincial level in China in 2020

Furthermore, the correlation analysis can provide more evidence for sustainable development. We compared *D*-values calculated in this study and sustainable competitiveness at the provincial level in China in 2020, as released by the Chinese Academy of Social Sciences (Table 2). It shows a significant positive correlation between these two indicators at the provincial level in China, 2020 (Fig. 6). Specifically, Gansu shows the lowest values of sustainable competitiveness score and *D*-values, indicating the low level of sustainable development potential and the coupling coordination degree of population aging and economic growth. Shanghai and Beijing present the highest values with great potential for sustainable development and high coupling coordination degrees of population aging and economic growth. Additionally, the scatter diagram of *D*-value and sustainable competitiveness can provide further information. The provinces in northwestern and southwestern China show lower levels of *D*-value and sustainable competitiveness. In contrast, Municipalities including Beijing, Shanghai, Tianjin, Chongqing, and some eastern provinces such as Zhejiang, Jiangsu, and Shandong present higher levels of *D*-value and sustainable competitiveness, indicating a distinct spatial difference.

## Discussion

Population aging and economic development are closely related. This paper selected two indicators to represent population aging and socio-economic development levels. Then, this study illustrated the spatial pattern and evolution of population aging and economic indicators in China from 2002 to 2020 by using standard deviational ellipse modeling. More importantly, the coupling coordination degree of population aging and economic development at the national and provincial levels were calculated and illustrated, and the spatial characteristics were investigated. Further, we can draw several key points by comparing existing studies and findings.

Previous studies found a similar movement trend of the economy and the total population in China as well as a southern direction movement of gravity centers [32, 33]. Further, a prior study revealed that the economic factors in China showed a southwestward movement, which is the reverse to the movement of population aging indicators [35]. Furthermore, the distances between the gravity centers of population aging and economic factors became smaller from 2002 to 2018 [35]. In this study, the evolution of the spatial distribution patterns of the population aging and economic indicators has been depicted by using SDE modeling, which offered more quantitative results than the method of gravity center. The standard deviation ellipses of GRP_pc_ moved southward, and those of EPR showed a reverse trend, consistent with the previous studies mentioned above. On the other hand, this study provided some new findings. The rotation of two indicators’ distribution orientation and directions show an opposite trend, and the orientation and directions of EPR are more evident than GRP_pc_ by using SDE modeling. Our findings further suggest that the difference in spatial distribution patterns of EPR and GRP_pc_ may widen in the following years, and the polarization of population aging may be more severe than economic development.

The coupling coordination degree of population aging and economic growth has been first evaluated at the national level and then at the provincial level in China. Our findings address the existing gap in knowledge about the coupling effect between population aging and economic growth by using CCDM. Beyond that, this study reveals an evident difference in space in the coordination states across the province in China. Several provinces with low D-values may reflect these two systems with low coupling states, such as Tibet, Qinghai, Yunnan, and Gansu. Prior studies demonstrated that the economic conditions in central-western China are relatively backward. However, the population aging’s degree and difference are increasing, which may increase the burden of medical services and welfare pensions for the aging population [60]. A previous study measured the vulnerability of population aging based on the indicators of the aging population ratio, health status, and GDP per capita, which found that the regions with the high vulnerability of population aging were located in northeastern, central, and western China [61]. Accordingly, our findings can offer implications for the demand for accelerated economic growth in the provinces with low *D*-values mentioned above, which may need economic growth to cope with the coming aging society. Furthermore, our findings provide further evidence of the relations between the coupling coordination degree and sustainable competitiveness. The provinces in northwestern, southwestern, and northeastern China both show lower levels of *D*-value and sustainable competitiveness, which is in line with the previous findings on the high vulnerability of population aging. These findings can help understand the challenges of population aging in space and provide a reference point for allocating resources and policymaking.

Several policy implications can be drawn based on our findings. First of all, since the coupling coordination degree of population aging and economic development displays significant spatial differentiation in China, future policymakers should fully consider regional differences in policy formulation and sustainable development. For instance, some northwestern provinces, such as Gansu, Ningxia, Xinjiang, and Qinghai, and some provinces in the southwestern part, such as Guizhou, Guangxi, and Yunnan, and northeastern provinces, such as Heilongjiang, present a low level of sustainable competitiveness and low levels of coupling coordination degree, which should be given policy priority in economic development when facing the coming aging society. In addition, the relations between the coupling coordination degree and sustainable competitiveness highlight the linking of population aging with sustainable competitiveness. Indeed, the need for linking population aging with the sustainable development concept is suggested as a future research topic by the Population Division of the United Nations [62] and some studies [22, 63]. Thus, population aging should be discussed in the context of other sustainable development challenges. For example, in regional development planning, population aging needs to be examined concerning other development challenges such as infrastructure development and innovation capability, essential parts of sustainable competitiveness [64]. For another example, several local policies have been conducted to attract the high-education level labor force to register their households (*Hukou*) in Zhengzhou City of Henan Province, Chengdu City of Sichuan Province, and Xi’an City of Shaanxi Province, which are pondering the impacts of rapid population aging on the local economic development [61]. Furthermore, the provinces with low levels of coupling coordination degree and sustainable competitiveness also showed relatively poor health status of the elderly, indicating a greater demand and pressure will be placed on the health and social care sectors. Therefore, some innovations in healthcare and social care can be recommended, such as telehealth, telecare, and telemedicine technologies and services to support aged people remaining independent for longer [65, 66], which are expected to help governments to improve sustainable competitiveness and to cope with the challenges of an increasingly aging population.

This paper has some limitations that need to improve in the future. Firstly, although economic factors and sustainable competitiveness are contemplated in this study, several social factors are excluded, such as health care resources, urbanization, and poverty levels. Thus, multi-dimensional analysis integrated with such indicators mentioned above can be conducted based on CCDM in the future. Secondly, for the following studies, a framework can be constructed in China for considering the physical environment implications of the growing elderly population, which can provide the potential for interactions between population aging and environmental exposures to produce adverse health effects in older adults [67]. Thirdly, this paper is only at the provincial level in China, and the following studies can be conducted across the prefectural and county levels. Comparing the outcomes of empirical analysis using a finer spatial scale can help gain a better understanding [68]. Fourthly, spatial modeling with urban-rural gaps can be performed in future studies. For instance, spatial disparities and vulnerability of population aging in rural areas are highlighted in China [61]. Meanwhile, the study in South Korea showed that the speed of population aging is linked to spatial inequalities in socio-economic development, and the old-age dependency ratio is relatively high in sparsely-populated rural areas [69]. Hence, the urban-rural and regional gap may play a role in the relations between population aging and economic growth, which should be investigated in-depth in the future.

## Conclusions

1. This paper illustrates the spatial pattern and evolution of population aging as well as economic indicators in China from 2002 to 2020 by using standard deviational ellipse modeling. As a result, the rotation of these two indicators’ distribution orientation and directions show an opposite trend, and the orientation and directions of EPR are more evident than GRP_pc_ by using SDE modeling. It further indicates that the difference in spatial distribution patterns of EPR and GRP_pc_ might widen in the following years, and the polarization of population aging may be more severe than China’s economic development.
2. The coupling coordination degree of population aging and economic growth is first evaluated at the national level in China, which shows an overall increasing trend from low coupling to moderate coupling from 2002 to 2020. Further, our findings reveal the spatial difference in the coupling and coordination degree across provinces. It shows that the eastern coastal areas were higher than China’s central and western areas. Moreover, significant positive autocorrelations can be found in the coupling coordination degree, indicating clustered spatial patterns.
3. Our findings demonstrate a significant positive correlation between coupling coordination degree and sustainable competitiveness at the provincial level in China. In addition, since the coupling coordination degree of population aging and economic development displays significant spatial differentiation in China, future policymakers should fully consider regional differences in policy formulation and sustainable development. Specifically, some provinces such as Gansu, Ningxia, Xinjiang, Qinghai, Guizhou, Guangxi, Yunnan, and Heilongjiang should be given policy priority in the regional development planning, and population aging needs to be examined concerning sustainable development challenges.

## Data Availability

All the data used in this study are from the national databases of China, which are publicly available, and unrestricted re-use is permitted.

## Abbreviations

CCDM: Coupling and coordination model
CV: Coefficient of variation
EPR: Elderly population rate
GRP_pc_: Per capita Gross Regional Product
SDE: Standard deviational ellipse

## References

[1] Luo Y, Su B, Zheng X (2021). Trends and challenges for population and health during population aging - China, 2015-2050. China CDC Wkly.

[2] Wan H, Yu F, Kolanowski A (2008). Caring for aging Chinese: lessons learned from the United States. J Transcult Nurs Off J Transcult Nurs Soc.

[3] Han Y, He Y, Lyu J, Yu C, Bian M, Lee L (2020). Aging in China: perspectives on public health. Glob Health J.

[4] Banister J, Zhang X (2005). China, economic development and mortality decline. World Dev.

[5] Bergh A, Nilsson T (2010). Good for living? On the relationship between globalization and life expectancy. World Dev.

[6] Alam MS, Islam MS, Shahzad SJH, Bilal S (2021). Rapid rise of life expectancy in Bangladesh: does financial development matter?. Int J Finance Econ.

[7] Lee S-H, Mason A, Park D. Overview: why does population aging matter so much for Asia? Population aging, economic growth, and economic security in Asia [Internet]. Manila, Philippines: Asian Development Bank; 2011. Report No.: ADB Economics Working Paper Series No. 284. Available from: http://ssrn.com/abstract=1960877

[8] Banister J, Bloom DE, Rosenberg L, Aoki M, Wu J (2012). Population aging and economic growth in China. Chin econ new transit.

[9] Asian Development Bank. Challenges and Opportunities of Population Aging in the People’s Republic of China [Internet]. Manila, Philippines: Asian Development Bank; 2014. Report No.: NO. 2014–3. Available from:www.adb.org

[10] Lindh T, Malmberg B (1999). Age structure effects and growth in the OECD, 1950–1990. J Popul Econ.

[11] Bloom DE, Canning D, Fink G (2010). Implications of population ageing for economic growth. Oxf Rev Econ Policy.

[12] Harper S (2014). Economic and social implications of aging societies. Science.

[13] Sheiner L (2014). The determinants of the macroeconomic implications of aging. Am Econ Rev.

[14] Bloom DE, Chatterji S, Kowal P, Lloyd-Sherlock P, McKee M, Rechel B (2015). Macroeconomic implications of population ageing and selected policy responses. Lancet.

[15] Wang S, Luo K, Liu Y, Zhang S, Lin X, Ni R (2015). Economic level and human longevity: spatial and temporal variations and correlation analysis of per capita GDP and longevity indicators in China. Arch Gerontol Geriatr.

[16] Lopreite M, Zhu Z (2020). The effects of ageing population on health expenditure and economic growth in China: a Bayesian-VAR approach. Soc Sci Med.

[17] Choi K-H, Shin S (2015). Population aging, economic growth, and the social transmission of human capital: an analysis with an overlapping generations model. Econ Model.

[18] Uddin GA, Alam K, Gow J (2016). Population age structure and savings rate impacts on economic growth: evidence from Australia. Econ Anal Policy.

[19] Kresl PK, Ietri D (2010). The aging population and the competitiveness of cities: benefits to the urban economy. Edward Elgar Publishing.

[20] Ono T, Maeda Y (2002). Sustainable development in an aging economy. Environ Dev Econ.

[21] Bai C, Lei X (2020). New trends in population aging and challenges for China’s sustainable development. China Econ J Routledge.

[22] Kudo S, Mutisya E, Nagao M (2015). Population aging: an emerging research agenda for sustainable development. Soc Sci Multidiscip Digit Publ Inst.

[23] Yu T (2013). China’s aging population and its spatial features in city areas (2000-2010). Urban plan Forum.

[24] Zhang K, Chen N (2014). Characteristics of spatial-temporal evolution in population aging and driving mechanism at county level in Fujian Province during 1990-2010. Prog Geogr.

[25] Li S, Cheng Y, Gao SY. The regional difference of population aging in Beijing-Tianjin-Hebei region. Popul Dev. 2017;23:2–12.

[26] Han X, Li J, Wang N (2018). Spatiotemporal evolution of Chinese ageing from 1992 to 2015 based on an improved Bayesian space-time model. BMC Public Health.

[27] Xu X, Zhao Y, Zhang X, Xia S (2018). Identifying the impacts of social, economic, and environmental factors on population aging in the Yangtze River Delta using the geographical detector technique. Sustainability.

[28] Wu Y, Song Y, Yu T (2019). Spatial differences in China’s population aging and influencing factors: the perspectives of spatial dependence and spatial heterogeneity. Sustainability.

[29] Qi Y, Yang Y, Jin F (2013). China’s economic development stage and its spatio-temporal evolution: a prefectural-level analysis. J Geogr Sci.

[30] Zhou Y, Li N, Wu W, Wu J (2014). Evolution of spatial-temporal pattern of county economic development in China during 1982-2010. Prog Geogr PROGRESS IN GEOGRAPHY.

[31] Fan J, Wang Y, Liang B (2019). The evolution process and regulation of China’s regional development pattern. Acta Geograph Sin.

[32] Xu Y, Li S (2005). Dynamic evolvement of the population and the social economy gravity center in China. Hum Geogr.

[33] Lian X (2007). Analysis on the space evolvement track of population gravity center, employment gravity center and economic gravity center. Popul J.

[34] Liang L, Chen M, Jiang Y, Luo X, Xian Y. Changes pattern in the population and economic gravity centers since the reform and opening up in China: the widening gaps between the south and north. J Clean Prod. 2021;127379.

[35] Man W, Wang S, Yang H (2021). Exploring the spatial-temporal distribution and evolution of population aging and social-economic indicators in China. BMC Public Health.

[36] Tomal M (2021). Evaluation of coupling coordination degree and convergence behaviour of local development: a spatiotemporal analysis of all polish municipalities over the period 2003–2019. Sustain Cities Soc.

[37] Gong J (2002). Clarifying the standard deviational ellipse. Geogr Anal.

[38] National Bureau of Statistics of China (2010). Tabulation on the 2010 population census.

[39] OECD (2019). Elderly population (indicator).

[40] United Nations Population Fund (2012). Ageing in the twenty-first century: a Celebration and a challenge.

[41] Roberts AW, Ogunwole SU, Blakeslee L, Rabe MA. The population 65 years and older in the United States. Am Community Surv Rep. 2016.

[42] United Nations (2020). World population prospects 2020.

[43] United Nations (2010). Gross regional product (GRP): an introduction [internet]. Department of Economic and Social Affairs, Statistics Division, United Nations.

[44] Bloom DE, Eggleston KN. The economic implications of population ageing in China and India: introduction to the special issue. J Econ Ageing. 2014; 4:1–7.

[45] Wang S (2020). Spatial patterns and social-economic influential factors of population aging: a global assessment from 1990 to 2010. Soc Sci Med.

[46] Wang Y, Wang G, Ni P. A study on the Masurement of sustainable competitiveness of Chinese cities. Econ Rev J. 2018:99–111.

[47] Peng J, Chen S, Lü H, Liu Y, Wu J (2016). Spatiotemporal patterns of remotely sensed PM2.5 concentration in China from 1999 to 2011. Remote Sens Environ.

[48] Huang D, Dong W, Wang Q (2021). Spatial and temporal analysis of human infection with the avian influenza a (H7N9) virus in China and research on a risk assessment agent-based model. Int J Infect Dis.

[49] Richter A, Ng KTW, Karimi N, Chang W (2021). Developing a novel proximity analysis approach for assessment of waste management cost efficiency in low population density regions. Sustain Cities Soc.

[50] Yang Z, Wu D, Wang D (2021). Exploring spatial path dependence in industrial space with big data: a case study of Beijing. Cities.

[51] Li Y, Li Y, Zhou Y, Shi Y, Zhu X (2012). Investigation of a coupling model of coordination between urbanization and the environment. J Environ Manag.

[52] Liu W, Jiao F, Ren L, Xu X, Wang J, Wang X (2018). Coupling coordination relationship between urbanization and atmospheric environment security in Jinan City. J Clean Prod.

[53] Wang J, Wang S, Li S, Feng K (2019). Coupling analysis of urbanization and energy-environment efficiency: evidence from Guangdong province. Appl Energy.

[54] Gan L, Shi H, Hu Y, Lev B, Lan H (2020). Coupling coordination degree for urbanization city-industry integration level: Sichuan case. Sustain Cities Soc.

[55] Ariken M, Zhang F, Chan N, weng, Kung H. (2021). Coupling coordination analysis and spatio-temporal heterogeneity between urbanization and eco-environment along the silk road Economic Belt in China. Ecol Indic.

[56] Wang Q, Han X (2021). Is decoupling embodied carbon emissions from economic output in Sino-US trade possible?. Technol Forecast Soc Change..

[57] Shang Y, Liu S (2021). Spatial-temporal coupling coordination relationship between urbanization and green development in the coastal cities of China. Sustainability.

[58] Moran PAP (1950). Notes on continuous stochastic phenomena. Biometrika.

[59] Cliff AD, Ord JK. Spatial processes: models & applications. Pion London. 1981;1–266.

[60] Chen M, Hao G (2014). Research on regional difference decomposition and influence factors of population aging in China. China Popul Resour Environ.

[61] Cheng Y, Gao S, Li S, Zhang Y, Rosenberg M (2019). Understanding the spatial disparities and vulnerability of population aging in China. Asia Pac policy stud. Wiley online. Library.

[62] UNDP (2014). Population ageing and sustainable development.

[63] Shetty P (2012). Grey matter: ageing in developing countries. Lancet.

[64] Balkyte A, Tvaronavičiene M (2010). Perception of competitiveness in the context of sustainable development: Facets of “sustainable competitiveness”. J Bus Econ Manag Taylor Francis.

[65] Flick C, Zamani ED, Stahl BC, Brem A (2020). The future of ICT for health and ageing: unveiling ethical and social issues through horizon scanning foresight. Technol Forecast Soc Change.

[66] Oderanti FO, Li F, Cubric M, Shi X (2021). Business models for sustainable commercialisation of digital healthcare (eHealth) innovations for an increasingly ageing population. Technol Forecast Soc Change.

[67] Geller AM, Zenick H (2005). Aging and the environment: a research framework. Environ health Perspect.

[68] Fotheringham AS, Brunsdon C, Charlton M (2000). Quantitative geography: perspectives on spatial data analysis.

[69] Yang HJ (2021). Visualizing spatial disparities in population aging in the Seoul metropolitan area. Environ Plan Econ Space.

